# Ribosome-Inactivating Proteins from *Salsola soda* L. and *Saponaria officinalis* L. Are Promising Candidates for Targeted Therapy of Colon Cancer

**DOI:** 10.3390/biomedicines14050981

**Published:** 2026-04-24

**Authors:** Francesco Biscotti, Sara Ragucci, Massimo Bortolotti, Federica Falà, Chiara Perrone, Nicola Landi, Andrea Bolognesi, Antimo Di Maro, Letizia Polito

**Affiliations:** 1 Department of Medical and Surgical Sciences—DIMEC, Alma Mater Studiorum, University of Bologna, Via San Giacomo 14, 40126 Bologna, Italy; francesco.biscotti2@unibo.it (F.B.); massimo.bortolotti2@unibo.it (M.B.); federica.fala@unibo.it (F.F.); chiara.perrone11@unibo.it (C.P.); andrea.bolognesi@unibo.it (A.B.); 2 Department of Environmental, Biological and Pharmaceutical Sciences and Technologies (DiSTABiF), University of Campania ‘Luigi Vanvitelli’, Via Vivaldi 43, 81100 Caserta, Italy; sara.ragucci@unicampania.it (S.R.); or nicola.landi@cnr.it (N.L.); 3 Institute of Crystallography, National Research Council of Italy, Via Vivaldi 43, 81100 Caserta, Italy

**Keywords:** colon cancer, immunoconjugates, immunotoxins, ribosome-inactivating proteins, *Salsola soda*, *Saponaria officinalis*, saporin-S6, sodin 5

## Abstract

**Background/Objectives**: Ribosome-inactivating proteins (RIPs) are plant-derived enzymes with potent cytotoxic activity, widely studied as anticancer agents, particularly as toxic payloads in immunoconjugates. Despite numerous encouraging results reported, their clinical application has been limited by their immunogenicity. RIPs from edible plants have been proposed as potentially more suitable candidates due to their possible improved tolerability. However, this aspect still requires validation in vivo in animal models. This study investigated the cytotoxic activity, mechanisms of action and translational potential of sodin 5 (a recently characterized type 1 RIP derived from the edible plant *Salsola soda* L.) in human colon cancer models, comparing it to the well-known type 1 RIP saporin-S6. **Methods**: The effects of sodin 5 and saporin-S6 on cell viability, cell death mechanisms and epithelial barrier integrity were assessed on HT29 and Caco-2 cell lines. Sodin 5 cross-reactivity with other anti-type 1 RIP sera was evaluated by ELISA. Finally, its structural characteristics were analyzed. **Results**: Sodin 5 showed a cytotoxic effect comparable to that of saporin-S6 in HT29 and Caco-2 colon cancer cells, with time- and concentration-dependent reductions in viability. Both type 1 RIPs disrupted the integrity of the intestinal epithelial barrier in mono- and co-culture models and predominantly activated the apoptotic pathway, without inducing necrosis. Sodin 5 exhibited limited immunological cross-reactivity and a conserved catalytic core, supporting its potential relevance as a therapeutic payload for intestinal cancer therapy. **Conclusions**: Our results indicate that sodin 5 possesses promising characteristics for anticancer applications, particularly in the treatment of intestinal malignancies, where local exposure and repeated administration are often required.

## 1. Introduction

Since ancient times, plant derivatives have played a primary role in traditional and folk medicine throughout the world for the treatment of several pathological conditions, mainly infectious diseases, inflammation and cancer [1,2,3]. Modern phytochemical and pharmacological studies have increasingly validated the therapeutic potential of these natural compounds [3,4,5,6]. Several plants used in traditional remedies have been found to contain ribosome-inactivating proteins (RIPs), a class of enzymes with rRNA N-glycosylase activity (EC 3.2.2.22) [7,8]. RIPs can remove a specific adenine in the 28S rRNA, thus irreversibly blocking protein translation. Besides rRNA, RIPs possess N-glycosylase activity also on other substrates; for this reason, RIP activity was also termed polynucleotide:adenosine glycosylase [9]. By acting on different substrates, RIPs can trigger various cell death pathways and eliminate even cells with altered proliferative or apoptotic mechanisms, thereby preventing the selection of RIP-resistant mutants [10]. These enzymes, which include both single-chain (type 1) and double-chain (type 2) RIPs, have been isolated and characterized in both medicinal [8,11] and edible plants [12,13]. Nowadays, the most promising pharmacological use of RIPs lies in the development of immunoconjugates, designed for the selective elimination of cells responsible for different pathological conditions [9,14,15]. Several type 1 RIPs, such as saporin-S6 from *Saponaria officinalis* L. seeds, have been thoroughly investigated as components of immunoconjugates for targeted cancer therapy [16,17]. The use of RIPs as toxic payloads in conjugates offers several advantages over conventional antineoplastic drugs, because toxins exert their action catalytically, do not induce multi-drug resistance and can eliminate cells in both proliferative and quiescent states. The main side effect reported in clinical trials with RIP-containing conjugates is immunogenicity. The generation of anti-RIP antibodies can represent a serious obstacle, which may limit repeated administration and reduce efficacy [18]. In this regard, edible plant-derived RIPs might potentially exhibit low immunogenicity and improved tolerability, although in vivo studies are so far lacking. *Salsola soda* L. is an edible plant well known for its potential as a source of bioactive compounds in addition to its nutritional value [19,20,21]. Among such bioactive compounds, type 1 RIPs called sodins have been found in various tissues of *S. soda*, including seeds, leaves and roots [22]. In previous studies, the major isoform sodin 5, isolated from seeds, has demonstrated promising biological properties [23].

The aim of the present study was to evaluate the cytotoxic activity and mechanism of action of sodin 5, assessing its translational potential relative to saporin-S6, one of the most widely characterized RIPs in oncological research. Specifically, we investigated the effects of sodin 5 on cell viability, evaluating triggered cell death pathways and epithelial barrier integrity in two human colon cancer-derived cell lines. In addition, the immunological properties of sodin 5 were assessed by determining its cross-reactivity with other anti-type 1 RIP sera. Finally, structural related features were analyzed to provide further insight into the biological behavior and therapeutic relevance of sodin 5 as a natural compound with potential application in intestinal cancer treatment.

## 2. Materials and Methods

### 2.1. Ribosome-Inactivating Proteins

Saporin-S6 was purified from the seeds of *S. officinalis* as previously described [24]. Sodin 5 was purified from *S. soda* seeds according to the procedure previously reported [22].

### 2.2. Cells

The human colon adenocarcinoma cell lines HT29 (lot number 300215-921) and Caco-2 (lot number 300137-220424) were obtained from Cytion (Eppelheim, Germany). The murine fibroblast 3T3 cell line was obtained from the departmental cell collection of the University of Bologna. Cells were cultured in Eagle’s minimum essential medium (Sigma Aldrich, Saint Louis, Dorset, MO, USA), supplemented as already described in [10]. Cells were maintained at 37 °C in a humidified atmosphere of 5% CO_2_ [10].

### 2.3. Cell Viability

Cell viability was determined using the CellTiter 96^®^ Aqueous One Solution Cell Proliferation Assay (Promega Corporation, Madison, WI, USA). HT29 and Caco-2 cells (3 × 10^3^/well) were seeded in 96-well microtiter plates (Falcon, Mexico City, Mexico). After 24 h, cells were incubated with scalar concentrations of sodin 5 or saporin-S6 (from 10^−10^ to 10^−5^ M). After 24, 48 and 72 h of incubation at 37 °C, the medium was removed and replaced with 100 μL/well of complete medium and 20 μL/well of kit. Following 2 h of incubation at 37 °C, absorbance was measured at 492 nm using the microtiter plate reader Multiskan EX (ThermoLab systems, Waltham, MA, USA). Each experiment was performed in triplicate. In order to obtain a comparative evaluation of cytotoxic effects of sodin 5 and saporin-S6, the effective concentration of RIP reducing 50% of viability (EC_50_) in both cell lines was calculated by linear regression analysis, applied to the quasi-linear portion of the dose–response curves.

### 2.4. Morphological Evaluation of RIP-Exposed Intestinal Cells

Intestinal cells treated with sodin 5 or saporin-S6 (10^−6^ M) for 24, 48 and 72 h were examined for morphological changes. Observations were carried out in 96-well plates using a phase-contrast microscope equipped with a digital camera (Nikon, Tokyo, Japan). Image acquisition was performed using X-Elit software, version 18.01.02 (Alexasoft, Florence, Italy).

### 2.5. Measurement of Transepithelial Electrical Resistance

Transepithelial electrical resistance (TEER) was evaluated on Caco-2 cell monoculture and on Caco-2/HT29 and Caco-2/3T3/collagen co-culture models. Cells (6.25 × 10^4^ Caco-2 cells/cm^2^ for monoculture experiments, 6.25 × 10^4^ Caco-2/HT29 cells/cm^2^ in a 9:1 ratio for co-culture experiments) were seeded in 24-well Transwell inserts (Corning, New York, NY, USA) with a 0.4 µm transparent polyester membrane. In the Caco-2/3T3/collagen co-culture model, 3T3 cells (0.78 × 10^4^ cells/cm^2^) were seeded on the apical compartment with 0.1 µg/mL of type 1 collagen (Corning) in complete medium. After 1 h at 37 °C, Caco-2 cells (6.25 × 10^4^/cm^2^) were seeded. In all experiments, complete medium (900 µL) was added to the basolateral compartment.

Once TEER values reached ≥500 Ω × cm^2^, cells were exposed to sodin 5 or saporin-S6 at a concentration of 10^−6^ M. TEER measurements were then recorded at 0, 8, 24, 32, 48 and 72 h using the voltohmmeter Millicel^®^ ERS 3.0 (Merck, Rahway, NJ, USA). A cell-free Transwell insert was used as the blank control. TEER values (Ω × cm^2^) were calculated using Equation (1):

TEER = (R − R_blanck_) × A (1)

where R is the resistance across the cell layer(s), R_blank_ corresponds to the resistance of the blank insert and A indicates the surface area of a Transwell membrane.

### 2.6. Assessment of Cell Death Pathways

The contribution of apoptosis, necroptosis and/or necrosis was investigated by flow cytometry analysis using the Annexin V-Enhanced Green Fluorescent Protein (EGFP)/Propidium Iodide (PI) detection kit (Biovision, Milpitas, CA, USA). This method allows for the discrimination between cells in necrosis (PI-positive and Annexin V-EGFP-negative), in late apoptosis/necroptosis (PI-positive and Annexin V-EGFP-positive) and in early apoptosis (PI-negative and Annexin V-EGFP-positive) [25]. Cells (4 × 10^5^) were seeded in 6-well plates (Corning), and after incubation with RIPs at 10^−6^ M concentration for 24 h, the cells were collected in cytofluorimeter tubes, pelleted at 400× *g* for 5 min, washed twice in cold 5 mM sodium phosphate buffer, pH 7.4, containing 0.14 M NaCl (PBS), pelleted and resuspended in 300 μL of binding buffer, containing Annexin V-EGFP (3 μL) and PI (3 μL). After 10 min of incubation in the dark at room temperature, cells were analyzed by flow cytometry CytoFLEX S (Beckman Coulter, Brea, CA, USA), using the CytExpert software (version 2.4.0.28, 2013).

The involvement of apoptosis and necroptosis was also indirectly evaluated using two death inhibitors, the pan-caspase inhibitor Z-VAD and the necroptosis inhibitor necrostatin-1 (NEC). The cytotoxic effects of sodin 5 and saporin-S6 were evaluated on HT29 and Caco-2 cells (3 × 10^3^/well in 96-well microtiter plates) pre-treated for 3 h with 100 μM Z-VAD or NEC, as previously reported [10]. The 3 h pre-treatment was carried out to ensure that any observed increase in cell survival could be attributed to the modulation of intracellular death mechanisms, rather than to potential extracellular interactions between the inhibitors and the RIPs that might directly affect toxin activity. The selected cell death inhibitor concentrations are the highest concentrations that were not toxic for HT29 and Caco-2 cells in preliminary assays. Following the 3 h incubation, the inhibitors were eliminated, and the cells were treated with sodin 5 or saporin-S6 (10^−6^ M) for 2 h. After washing with 100 μL/well of PBS, cells were maintained for a further 24 h in complete medium, and cell viability was then determined as mentioned above.

### 2.7. Sodin 5 Immunological Properties

ELISA assay was performed in a 96-well plate (Sarstedt, Nümbrecht, Germany), coated with 2 μg per well of sodin 5 in 100 μL of 50 mM carbonate buffer pH 9.0, with 15 mM sodium carbonate and 35 mM sodium bicarbonate. After overnight incubation at 4 °C to allow RIP adhesion, each well was washed 5 times with 200 μL PBS/Tween (137 mM NaCl, 1.5 mM KH_2_PO_4_, 8 mM Na_2_HPO_4_, 0.05% (*v*/*v*) Tween 20), and 200 μL/well of 0.5 mg/mL bovine serum albumin was added to saturate well charges. After 1 h of incubation at 37 °C and a further 5 washes with PBS/Tween, reciprocal serum dilutions (from 1:400 to 1:409,600) in PBS/Tween were added. After incubation at 37 °C for 3 h and 5 washes with PBS/Tween, an anti-rabbit secondary antibody conjugated to alkaline phosphatase (Merck, Burlington, MA, USA) was added in 100 μL/well at 1:7000 dilution, and the plate was incubated at 37 °C for 1 h. After further 5 washes with PBS/Tween, 100 μL of 1 mg/mL 4-nitrophenyl phosphate disodium (Merck), dissolved in a buffer containing 1 M diethanolamine, 0.5 M MgCl_2_ × 6H_2_O, and 3 mM NaN_3_, was added. Absorbance was measured at 405 nm with the Multiskan EX microtiter plate reader (ThermoLabsystem, Helsinki, Finland). Results are expressed as mean ± standard deviations of three independent experiments, each carried out in triplicate.

### 2.8. Amino Acid Analysis of Sodin 5

Amino acid analyses were performed as previously reported [26] by using a Biochrom30+ amino acid analyzer (Biochrom, Cambridge, UK), equipped with a post-column ninhydrin derivatization system. In addition, to detect cysteine, sodin 5 was subjected to oxidation with performic acid. Briefly, 100 μg of protein was hydrolyzed in a glass tube and 400 µL of performic acid was added. After incubation at 0 °C for 60 min, 200 µL of cold HBr was added. Samples were dried in a desiccator, rinsed with water, and then the hydrolyzed samples were analyzed.

### 2.9. Statistical Analysis

Statistical analyses were executed using XLSTAT-Pro Software, version 6.1.9, 2003 (Addisoft, Inc., Brooklyn, NY, USA). The results are presented as means ± S.D. of two (flow cytometry experiments) or three (cell viability, TEER and ELISA experiments) independent experiments, each carried out in triplicate. For comparisons involving multiple groups (e.g., different concentrations or treatments at a given timepoint), one-way ANOVA followed by Bonferroni post hoc test or Dunnett’s test (when comparing multiple treatments to a single control) were used. For comparisons between two groups, the Mann–Whitney U test was applied. Time-course experiments were analyzed by comparing each timepoint to the corresponding control condition.

## 3. Results

### 3.1. Sodin 5 and Saporin-S6 Show Similar Cytotoxicity on Colon Cancer-Derived Cells

The cytotoxic effect of type 1 RIPs was evaluated by their ability to reduce cell viability on two colon cancer-derived cell lines, HT29 and Caco-2, in concentration- and time-response experiments. Cell viability was assessed after 24, 48 and 72 h of incubation with scalar concentrations of RIPs, comparing sodin 5 cytotoxicity with that of saporin-S6.

**Figure 1:**
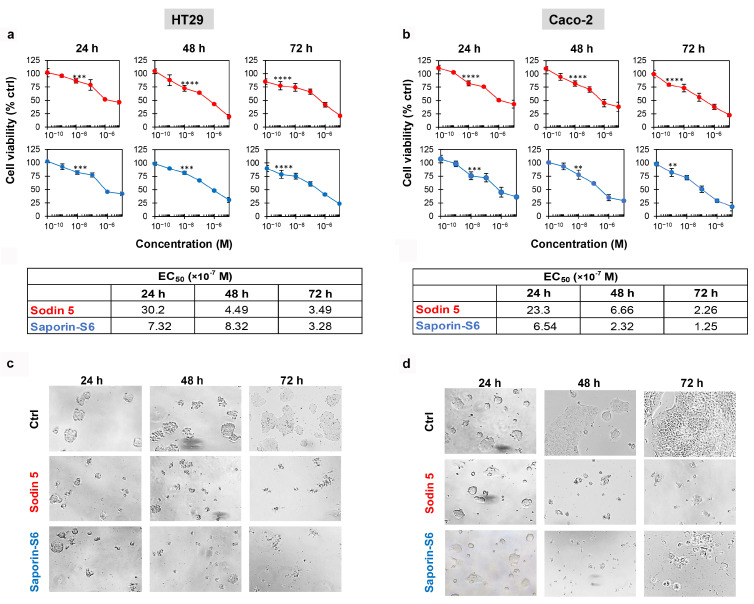
Effect of sodin 5 and saporin-S6 on cell viability in HT29 and Caco-2 cells. HT29 (**a**) and Caco-2 (**b**) cell viabilities were evaluated at 24, 48 and 72 h after exposure to the indicated concentrations of sodin 5 (red lines) and saporin-S6 (blue lines). Cell viability was measured using a colorimetric assay based on MTS reduction. The mean results ± S.D. are reported, representing the percentage of control values. Data were analyzed by ANOVA/Bonferroni test, followed by a comparison with Dunnett’s test (confidence range 95%; ** *p* < 0.01, *** *p* < 0.001, **** *p* < 0.0001, versus untreated controls). Asterisks indicate the first concentration at which a statistically significant reduction in cell viability is observed compared to controls. The morphology of HT29 (**c**) and Caco-2 (**d**) cells was evaluated at the indicated times for control (Ctrl) and for treated cells (sodin 5 or saporin-S6 at 10^−6^ M concentration). Images were taken using phase-contrast microscopy (total magnification 100×).

In HT29 and Caco-2 cells, a significant reduction in cell viability (Figure 1a,b) was observed for both sodin 5 (red line) and saporin-S6 (blue line) starting from 10^−8^ M concentration, after 24 and 48 h. At 72 h, a highly significant reduction in cell viability (*p* ≤ 0.0001) for both RIPs was already evident at 10^−9^ M. The comparison of EC_50_s demonstrates that sodin 5 and saporin-S6 share a very similar cytotoxic profile on both cell lines. Although saporin-S6 exhibited greater efficacy than sodin 5 after 24 h of exposure, with a difference of approximately one order of magnitude, the EC_50_ values obtained in both cellular models after 72 h of treatment were of the same order of magnitude (10^−7^ M), indicating that the long-term effects of the two RIPs are fully comparable (Table 1). Moreover, within the time frame considered in our experimental design (24–72 h), the decline in cell viability curves was highly comparable in both cell lines, suggesting that the majority of the cytotoxic effect is already achieved within the first 24 h of RIP exposure. Consistent with the viability data, both sodin 5 and saporin-S6 induced morphological changes, mainly characterized by cell shrinkage, as shown in Figure 1c,d.

### 3.2. Epithelial Barrier Integrity Is Compromised by Sodin 5 and Saporin-S6 in Both Monoculture and Co-Culture Models

The impact of sodin 5 and saporin-S6 on the integrity of the epithelial barrier was evaluated by measuring changes in TEER at different timepoints across three in vitro models: Caco-2 monoculture, Caco-2/HT29 and Caco-2/3T3/collagen co-cultures.

**Figure 2:**
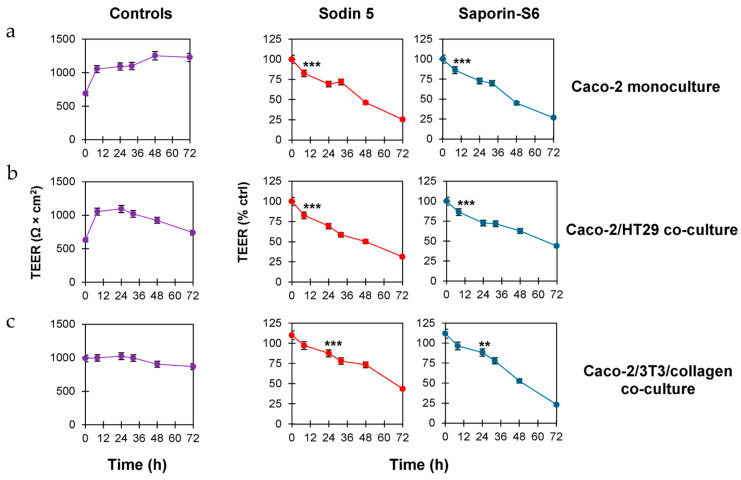
Time-dependent TEER changes across Caco-2 monoculture, Caco-2/HT29 and Caco-2/3T3/collagen co-culture models. Caco-2 monoculture (**a**), Caco-2/HT29 (**b**) and Caco-2/3T3/collagen (**c**) co-culture models were established by seeding cells (6.25 × 10^4^ Caco-2 cells/cm^2^ for monoculture experiments, 6.25 × 10^4^ Caco-2/HT29 cells/cm^2^, in a 9:1 ratio, for co-culture experiments) on 24-well Transwell inserts with 0.4 µm transparent polyester membrane. In the Caco-2/3T3/collagen co-culture model, 3T3 cells (0.78 × 10^4^ cells/cm^2^) were seeded on the apical compartment with 0.1 µg/mL of type 1 collagen in complete medium. After 1 h at 37 °C, Caco-2 cells (6.25 × 10^4^/cm^2^) were seeded. In all experiments, complete medium (900 µL) was added to the basolateral compartment. All cultures were maintained about 10 days, replacing culture medium every 2–3 days. When TEER reached values ≥ 500 Ω × cm^2^ (about 10 days), cell monolayers were treated with sodin 5 or saporin-S6 at 10^−6^ M concentration. TEER values were recorded at 0, 8, 24, 32, 48 and 72 h after sodin 5 or saporin-S6 intoxication. Results are expressed as mean ± SD of three independent experiments, each conducted in triplicate. Data were analyzed by ANOVA/Bonferroni test, followed by a comparison with Dunnett’s test (confidence range 95%; ** *p* < 0.01, *** *p* < 0.001 versus untreated controls). Asterisks indicate the first timepoint at which a statistically significant reduction in TEER is observed compared to controls.

Our data, reported in Figure 2, demonstrate that sodin 5 and saporin-S6 impair the epithelial barrier integrity in a time-dependent manner. Moreover, in the three different in vitro models, the TEER values were similarly affected by sodin 5 and saporin-S6.

In the Caco-2 monoculture model, both sodin 5 and saporin-S6 significantly reduced TEER values (*p* < 0.001) after 8 h at 10^−6^ M concentration, with values about 1.2-fold lower than controls (Figure 2a). This reduction became more pronounced at 24, 48 and 72 h, reaching TEER values about 1.4-, 2.2- and 4.0-fold lower than controls, respectively. A similar trend of TEER reduction was observed in the Caco-2/HT29 co-culture model. However, the difference in TEER values between treated and control cells was slightly less evident compared to the monoculture model (Figure 2b). The results obtained in the more complex co-culture model of Caco-2/3T3/collagen further confirm the barrier-disrupting effect of both RIPs that caused a significant reduction in TEER values over time (Figure 2c).

### 3.3. Sodin 5 Induces Apoptosis and Necroptosis but Not Necrosis, Exhibiting Cytotoxic Potency Comparable to That of Saporin-S6

**Figure 3:**
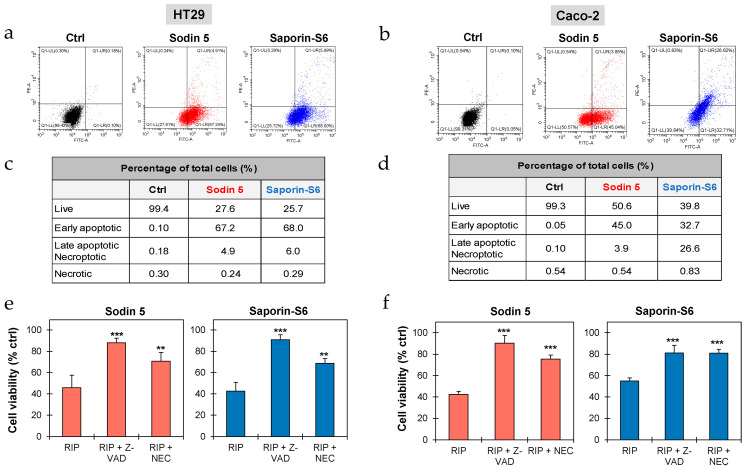
Evaluation of cell death mechanisms triggered by sodin 5 and saporin-S6. HT29 (**a**) and Caco-2 (**b**) cells (4 × 10^5^/well) were seeded in 6-well plates and cultured for 24 h in the absence or presence of sodin 5 or saporin-S6 at 10^−6^ M concentration. Apoptosis, necroptosis and necrosis were evaluated through flow cytometry analysis. Representative plots of Annexin V-EGFP (FITC channel)/PI (PE channel) staining of HT29 and Caco-2 cells are shown. Cell populations were characterized based on staining: necrotic cells (PI-positive and EGFP-negative) are in the upper left quadrant; late apoptotic and/or necroptotic cells (PI-positive and EGFP-positive) are in the upper right quadrant; early apoptotic cells (PI-negative and EGFP-positive) are in the lower right quadrant. The plots are representative of two independent experiments, each conducted in triplicate. The tables report the percentages of live, early apoptotic, late apoptotic and/or necroptotic and necrotic HT29 (**c**) and Caco-2 (**d**) cells after sodin 5 or saporin-S6 treatment. The protective effect of cell death inhibitors was evaluated on HT29 (**e**) and Caco-2 (**f**) cells (3 × 10^3^/well), treated with 10^−6^ M sodin 5 or saporin-S6. The inhibitors Z-VAD (apoptosis) or necrostatin-1 (NEC, necroptosis) were added at 100 μM concentration 3 h before the RIP treatment. Cells were then treated for 2 h with sodin 5 or saporin-S6 and further incubated for 24 h in complete medium. Cell viability was evaluated using a colorimetric assay based on MTS reduction. The results are expressed as means ± S.D. of three independent experiments, each conducted in triplicate. Data were analyzed by the Mann–Whitney U test (confidence range 95%; ** *p* < 0.01, *** *p* < 0.001 versus RIP-treated samples).

The involvement of apoptosis, necroptosis and/or necrosis in sodin 5- and saporin-S6-treated cells was evaluated through Annexin V-EGFP/PI double staining, followed by cytofluorimetric analysis. After 24 h of intoxication with RIPs at 10^−6^ M concentration, more than 67% of HT29 cells were in early-stage apoptosis, whereas about 45% and 33% of Caco-2 cells were in early-stage apoptosis after treatment with sodin 5 and saporin-S6, respectively. Only a small fraction of cells was found to undergo late apoptosis and/or necroptosis, accounting for approximately 5% and 6% of HT29 cells treated with sodin 5 and saporin-S6, respectively, and about 4% of Caco-2 cells treated with sodin 5. In contrast, in Caco-2 cells exposed to saporin-S6, the proportion of cells in late apoptosis and/or necroptosis increased markedly, reaching approximately 27%. Notably, both RIPs did not significantly induce necrosis in treated intestinal cells (Figure 3a–d).

Further experiments were conducted to elucidate the involvement of apoptosis and necroptosis in the RIP-induced cytotoxicity. Cells were pre-treated for 3 h with the pan-caspase inhibitor Z-VAD and the necroptosis inhibitor NEC. As shown in Figure 3e,f, the addition of cell death inhibitors significantly increased the cell viability of sodin 5- and saporin-S6-treated cells. This protective effect was observed in both cell lines, confirming that apoptosis and necroptosis are involved in cytotoxic mechanisms triggered by both RIPs.

### 3.4. Sodin 5 Does Not Cross-React with Anti-Saporin-S6 and Other Anti-Type 1 RIP Sera

**Figure 4:**
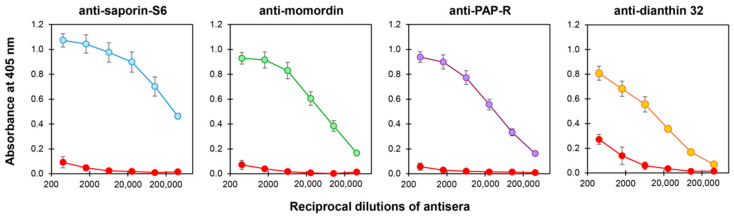
Immunological cross-reactivity of sodin 5 with anti-type 1 RIP sera evaluated through enzyme-linked immunosorbent assay (ELISA). The panels show the cross-reactivity curves of sodin 5 (red lines) and the specific RIP (saporin-S6, blue line; momordin, green line; PAP-R, purple line and dianthin 32, orange line) towards each anti-RIP serum. Reactivity is expressed as absorbance at 405 nm as a function of the reciprocal dilutions of the antiserum. Results are expressed as mean ± SD of three independent experiments, each conducted in triplicate.

Sodin 5 cross-reactivity with other type 1 RIPs, i.e., saporin-S6, momordin (from *Momordica charantia* L. seeds), PAP-R (from *Phytolacca americana* L. roots) and dianthin 32 (from *Dianthus caryophyllus* L. leaves), was investigated by the specific anti-RIP sera. Sodin 5 showed no significant cross-reactivity with anti-saporin-S6, anti-momordin or anti-PAP-R sera, and only very low reactivity was observed with anti-dianthin serum. These results suggest that sodin 5 possesses surface epitopes distinct from those of such well-known RIPs (Figure 4).

### 3.5. Sodin 5 Shares a Conserved Catalytic Core with Saporin-S6

**Figure 5:**
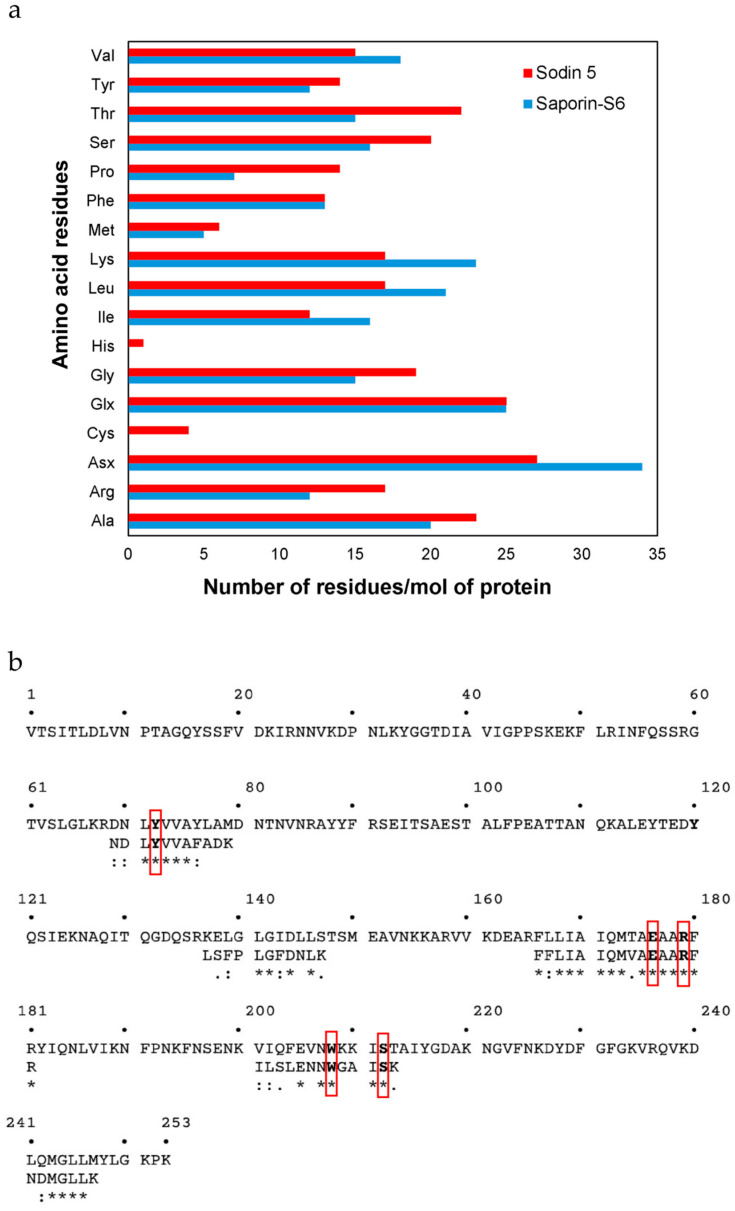
Structural analysis of sodin 5 compared to saporin-S6. (**a**) Amino acid composition of sodin 5 and saporin-S6. The histograms show the comparison of the amino acid content for sodin 5 (red bars) and saporin-S6 (blue bars), expressed as the number of residues per mole of protein. The three-letter amino acid code is used. (**b**) Amino acid sequence alignment of sodin 5 with saporin-S6. The panel shows the alignment of selected sodin 5 tryptic peptides against the amino acid sequence of saporin-S6 (Uniprot accession number: P20656). Conserved amino acid residues found in the active site of RIPs are indicated in bold and red boxes. Similarity is denoted by identical residues (∗), conserved substitutions (:) and semi-conserved substitutions (.).

Moreover, in order to verify other possible structural similarities/differences between sodin 5 and saporin-S6 (Uniprot accession number: P20656), the amino acid composition of sodin 5 was determined and compared with that of saporin-S6, taking into account the difficulties in obtaining information on the primary structure of sodin 5, which has the N-terminal amino acid residue blocked [23]. The data reported in Figure 5a show several differences in the content of amino acids per mole of protein. In addition, the analysis revealed the presence of four cysteines that are not present in saporin-S6. On the other hand, the content of Arg and Lys, as well as Phe and Tyr per mole of protein determined in sodin 5 was 34 (17 Arg + 17 Lys) and 27 (14 Phe + 13 Tyr), similar to that of saporin-S6 (35 (12 Arg + 23 Lys) and 25 (13 Phe + 12 Tyr)). Finally, the amino acid sequences of some tryptic peptides previously obtained by high-resolution nano-LC-tandem mass spectrometry [23] were overlapped with the amino acid sequence of saporin-S6 (Figure 5b). It is evident that the five tryptic peptide sequences of sodin 5 showed a high identity/similarity with some amino acid regions of saporin-S6. In particular, the analysis highlights the high similarity of the catalytic region (position 165–181), where most of the amino acids, involved in the catalytic activity (i.e., Glu176, Arg179) or in the interaction with the ribosomal target adenine (i.e., Tyr72, Trp208, Ser212), are conserved [16].

## 4. Discussion

A crucial and continuous challenge in the development of highly effective immunotherapeutic strategies is the identification of new compounds that can specifically target pathological or aberrant cells to achieve therapeutic efficacy while minimizing collateral damage and adverse effects [27,28,29]. In fact, the main adverse effect reported in clinical trials involving RIP-containing conjugates is immunogenicity, as the induction of anti-RIP antibodies may constitute a significant limitation by hindering repeated administrations and potentially reducing therapeutic efficacy through immune-mediated neutralization or accelerated clearance of the toxin component [30,31].

In this work, we investigated the cytotoxic effect of two type 1 RIPs, sodin 5 from the edible plant *S. soda* and saporin-S6 from *S. officinalis*, on two human colon cancer-derived cell lines. RIPs derived from edible plant species may represent promising therapeutic agents for the treatment of intestinal cancers. Furthermore, their derivation from edible plant sources may have implications for tolerability, given that dietary proteins are typically linked to mechanisms of immune tolerance and, in certain contexts, they can actively influence immune responses. Nevertheless, it remains to be established whether these properties result in decreased immunogenicity in therapeutic applications [32].

Sodin 5 efficiently reduced cell viability in concentration- and time-response experiments on both tested intestinal cell lines. The comparable EC_50_ values demonstrate cytotoxic effects similar to those of saporin-S6, the most widely utilized type 1 RIP in immunoconjugate development. The overall strong cytotoxic effect of sodin 5 on HT29 and Caco-2 intestinal cancer cell lines is highly encouraging.

Sodin 5 and saporin-S6 affected the integrity of the epithelial barrier in Caco-2 monoculture and Caco-2/HT29 co-culture models. Both RIPs markedly decreased TEER values, suggesting that sodin 5 may weaken the epithelial barrier tight junctions by increasing paracellular permeability. Reduction in TEER values was observed even in the more complex Caco-2/3T3/collagen model, which better mimics the in vivo environment. From a pathophysiological perspective, compounds capable of altering intestinal barrier integrity may raise safety concerns, given the importance of intact mucosa for maintaining intestinal homeostasis and preventing the translocation of microorganisms, toxins and pro-inflammatory mediators [33,34,35]. However, within a controlled therapeutic framework, especially for oncological treatments, such barrier modulation may also represent a favorable aspect, provided that it is targeted, reversible and temporally limited. In fact, when applied in an anticancer setting, this effect on barrier integrity could facilitate the access of cytotoxic agents within the poorly reachable tumor mass, thus affecting the therapeutic efficacy [36,37].

The combined use of Annexin V/PI staining in cytofluorimetric experiments is widely and commonly adopted as a first-level approach to distinguish between apoptotic and necrotic cell death pathways [38,39,40]. Our experiments on cell death mechanisms revealed a predominant role for apoptosis in HT29 and Caco-2 cells intoxicated with the two type 1 RIPs, with a significant percentage of cells undergoing apoptosis without necrosis involvement. However, the concomitant involvement of necroptosis alongside apoptosis was demonstrated through experiments employing inhibitors of apoptosis (Z-VAD) and necroptosis (NEC). In both cell lines, pre-treatment with these inhibitors significantly rescued cells from death induced by saporin-S6/sodin 5. Notably, our study provides the first evidence of necroptosis participation in intestinal cells exposed to type 1 RIPs.

The predominant involvement of apoptosis could represent a significant advantage in the future clinical application of sodin 5-containing immunotoxins, as the induction of regulated apoptotic cell death pathways, rather than necrosis, would attenuate the onset of pro-inflammatory responses [41]. However, the concomitant involvement of necroptosis observed in this study introduces an additional layer of complexity. Although necroptosis is a regulated form of cell death, it is intrinsically pro-inflammatory due to the release of molecular patterns associated with intracellular damage. In the context of intestinal tissues, this aspect is particularly relevant, as necroptosis has been implicated in the pathogenesis of chronic inflammatory conditions of the intestine. In particular, its activation may contribute to the development and exacerbation of chronic inflammatory bowel diseases, as well as to the compromised integrity of the intestinal barrier [42].

Taken together, these considerations highlight the dual nature of the cell death mechanisms triggered by sodin 5 and saporin-S6, underlining the need for further investigations to better define the balance between therapeutic efficacy and potential pro-inflammatory effects.

The main issue limiting the effectiveness of RIP-containing immunoconjugates is represented by immunogenicity [17]. Although the use of humanized carriers has substantially reduced anti-carrier immune responses, immune recognition of the toxin component continues to represent a critical issue in immunotoxin-based approaches. A potential strategy to mitigate anti-toxin antibody formation relies on the sequential use of immunotoxins containing distinct and non-cross-reactive RIPs. Thus, the evaluation of sodin 5 cross-reactivity with other type 1 RIPs represents a key aspect of the present study. The immunological analysis revealed low cross-reactivity between sodin 5 and sera against saporin-S6 and some other type 1 RIPs. These findings support the hypothesis that sodin 5-based immunotoxins may be suitable for use in alternating treatment regimens with immunotoxins containing different type 1 RIPs, potentially minimizing the risk of hypersensitivity reactions associated with the toxic moiety.

The similarities between sodin 5 and saporin-S6 were further clarified by structural analysis. Although the N-terminal amino acid residue of sodin 5 is blocked [23], making it difficult to determine its primary structure, the amino acid composition analysis showed variations in the number of amino acids of the two RIPs, most notably the presence of four cysteine residues in sodin 5 that are not present in saporin-S6 and some other type 1 RIPs. However, high similarity was revealed by aligning the tryptic peptides of sodin 5 with the sequence of saporin-S6, especially in the catalytic region (positions 165–181). Sodin 5 cytotoxic potential depends on the conservation of active site residues (Glu176 and Arg179) and of those involved in ribosomal adenine interaction (Tyr72, Trp208 and Ser212).

## 5. Conclusions

In conclusion, sodin 5, a type 1 RIP isolated from the edible plant *S. soda*, exhibits significant cytotoxic activity against human colon cancer-derived cell lines, mainly by activating apoptosis. These results highlight that sodin 5 is a naturally occurring compound with promising characteristics for anticancer applications, particularly in the treatment of intestinal malignancies, where local exposure and repeated administration are often required. The potential of sodin 5 as a candidate for immunotherapeutic development is further strengthened by its low immunological cross-reactivity with saporin-S6 and other type 1 RIPs. Our findings support the utility of edible plant-derived RIPs as promising therapeutic agents for immunotoxin construction. However, further preclinical evaluation is required, evaluating the absence of specific toxicity towards non-target cells and in vivo immunogenicity.

## Figures and Tables

**Table 1:** Effective concentrations of sodin 5 and saporin-S6 reducing 50% of viability (EC_50_) in HT29 and Caco-2 cell lines at 24, 48 and 72 h of intoxication. EC_50_ values were calculated by linear regression analysis of concentration–effect curves.

Cell Line	RIP	EC_50_ (M)		
		24 h	48 h	72 h
HT29	Sodin 5	3.02 × 10^−6^	4.49 × 10^−7^	3.49 × 10^−7^
	Saporin-S6	7.32 × 10^−7^	8.32 × 10^−7^	3.28 × 10^−7^
Caco-2	Sodin 5	2.33 × 10^−6^	6.66 × 10^−7^	2.26 × 10^−7^
	Saporin-S6	6.54 × 10^−7^	2.32 × 10^−7^	1.25 × 10^−7^

## Data Availability

The original contributions presented in this study are included in this article. Further inquiries can be directed at the corresponding authors.

## Abbreviations

The following abbreviations are used in this manuscript:

EC_50_	Effective concentration reducing cell viability by 50%
EGFP	Enhanced Green Fluorescent Protein
ELISA	Enzyme-linked immunosorbent assay
NEC	Necrostatin-1
PBS	Phosphate-buffered saline
PI	Propidium iodide
RIP	Ribosome-inactivating protein
SDS-PAGE	Sodium dodecyl sulfate polyacrylamide gel electrophoresis
TEER	Transepithelial electrical resistance

## References

[1] Chaachouay N., Zidane L. (2024). Plant-derived natural products: A source for drug discovery and development. Drugs Drug Candidates.

[2] Gahtori R., Tripathi A.H., Kumari A., Negi N., Paliwal A., Tripathi P., Joshi P., Rai R.C., Upadhyay S.K. (2023). Anticancer plant-derivatives: Deciphering their oncopreventive and therapeutic potential in molecular terms. Futur. J. Pharm. Sci..

[3] Latif R., Nawaz T. (2025). Medicinal plants and human health: A comprehensive review of bioactive compounds, therapeutic effects, and applications. Phytochem. Rev..

[4] Dai L., Li Z., Chen D., Jia L., Guo J., Zhao T., Nordlund P. (2020). Target identification and validation of natural products with label-free methodology: A critical review from 2005 to 2020. Pharmacol. Ther..

[5] Yang Y., Jiang B., Shi L., Wang L., Yang Y., Li Y., Zhang Y., Zhu Z., Zhang X., Liu X. (2025). The potential of natural herbal plants in the treatment and prevention of non-small cell lung cancer: An encounter between ferroptosis and mitophagy. J. Ethnopharmacol..

[6] Li Q.Y., Wang C.B., Liu W.J., Bu X.S., Zheng Y.D., Meng X.H., Shi X.F., Yang J.L. (2026). Advances in natural medicinal plant-based interventions against hypoxia-related neuroinflammation. J. Ethnopharmacol..

[7] Polito L., Bortolotti M., Maiello S., Battelli M.G., Bolognesi A. (2016). Plants producing ribosome-inactivating proteins in traditional medicine. Molecules.

[8] Schrot J., Weng A., Melzig M.F. (2015). Ribosome-inactivating and related proteins. Toxins.

[9] Bolognesi A., Bortolotti M., Maiello S., Battelli M.G., Polito L. (2016). Ribosome-inactivating proteins from plants: A historical overview. Molecules.

[10] Biscotti F., Bortolotti M., Falà F., Di Maro A., Bolognesi A., Polito L. (2025). Ricin toxicity to intestinal cells leads to multiple cell death pathways mediated by oxidative stress. Toxins.

[11] de Virgilio M., Lombardi A., Caliandro R., Fabbrini M.S. (2010). Ribosome-inactivating proteins: From plant defense to tumor attack. Toxins.

[12] Barbieri L., Polito L., Bolognesi A., Ciani M., Pelosi E., Farini V., Jha A.K., Sharma N., Vivanco J.M., Chambery A. (2006). Ribosome-inactivating proteins in edible plants and purification and characterization of a new ribosome-inactivating protein from *Cucurbita moschata*. Biochim. Biophys. Acta.

[13] Sharma A., Gupta S., Sharma N.R., Paul K. (2023). Expanding role of ribosome-inactivating proteins: From toxins to therapeutics. IUBMB Life.

[14] Setayesh-Mehr Z., Poorsargol M. (2021). Toxic proteins application in cancer therapy. Mol. Biol. Rep..

[15] Wang S., Li Z., Li S., Di R., Ho C., Yang G. (2016). Ribosome-inactivating proteins (RIPs) and their important health promoting property. RSC Adv..

[16] Giansanti F., Flavell D.J., Angelucci F., Fabbrini M.S., Ippoliti R. (2018). Strategies to improve the clinical utility of saporin-based targeted toxins. Toxins.

[17] Polito L., Bortolotti M., Mercatelli D., Battelli M.G., Bolognesi A. (2013). Saporin-S6: A useful tool in cancer therapy. Toxins.

[18] Rust A., Partridge L.J., Davletov B., Hautbergue G.M. (2017). The use of plant-derived ribosome inactivating proteins in immunotoxin development: Past, present and future generations. Toxins.

[19] Atzori G., Guidi Nissim W., Mancuso S., Palm E. (2022). Intercropping salt-sensitive *Lactuca sativa* L. and salt-tolerant *Salsola soda* L. in a saline hydroponic medium: An agronomic and physiological assessment. Plants.

[20] Centofanti T., Bañuelos G. (2015). Evaluation of the halophyte *Salsola soda* as an alternative crop for saline soils high in selenium and boron. J. Environ. Manage.

[21] Bañuelos G.S., Centofanti T., Zambrano M.C., Vang K., Lone T.A. (2022). *Salsola soda* as selenium biofortification crop under high saline and boron growing conditions. Front. Plant Sci..

[22] Landi N., Ragucci S., Citores L., Clemente A., Hussain H.Z.F., Iglesias R., Ferreras J.M., Di Maro A. (2022). Isolation, characterization and biological action of type-1 ribosome-inactivating proteins from tissues of *Salsola soda* L. Toxins.

[23] Novak Babič M., Ragucci S., Leonardi A., Pavšič M., Landi N., Križaj I., Gunde-Cimerman N., Sepčić K., Di Maro A. (2024). Biocontrol potential of sodin 5, type 1 ribosome-inactivating protein from *Salsola soda* L. seeds. Biomolecules.

[24] Stirpe F., Gasperi-Campani A., Barbieri L., Falasca A., Abbondanza A., Stevens W.A. (1983). Ribosome-inactivating proteins from the seeds of *Saponaria officinalis* L. (soapwort), of *Agrostemma githago* L. (corn cockle) and of *Asparagus officinalis* L. (asparagus), and from the latex of *Hura crepitans* L. (sandbox tree). Biochem. J..

[25] Pietkiewicz S., Schmidt J.H., Lavrik I.N. (2015). Quantification of apoptosis and necroptosis at the single cell level by a combination of Imaging Flow Cytometry with classical Annexin V/propidium iodide staining. J. Immunol. Methods.

[26] Chambery A., Di Maro A., Parente A. (2008). Primary structure and glycan moiety characterization of PD-Ss, type 1 ribosome-inactivating proteins from *Phytolacca dioica* L. seeds, by precursor ion discovery on a Q-TOF mass spectrometer. Phytochemistry.

[27] Fabbrini M.S., Katayama M., Nakase I., Vago R. (2017). Plant ribosome-inactivating proteins: Progesses, challenges and biotechnological applications (and a few digressions). Toxins.

[28] Rui R., Zhou L., He S. (2023). Cancer immunotherapies: Advances and bottlenecks. Front. Immunol..

[29] Singh K., Gupta J.K., Chanchal D.K., Shinde M.G., Kumar S., Jain D., Almarhoon Z.M., Alshahrani A.M., Calina D., Sharifi-Rad J. (2025). Natural products as drug leads: Exploring their potential in drug discovery and development. Naunyn Schmiedebergs Arch. Pharmacol..

[30] Kim J.S., Jun S.Y., Kim Y.S. (2020). Critical issues in the development of immunotoxins for anticancer therapy. J. Pharm. Sci..

[31] Akbari B., Farajnia S., Ahdi Khosroshahi S., Safari F., Yousefi M., Dariushnejad H., Rahbarnia L. (2017). Immunotoxins in cancer therapy: Review and update. Int. Rev. Immunol..

[32] Blum J.E., Kong R., Schulman E.A., Chen F.M., Upadhyay R., Romero-Meza G., Littman D.R., Fischbach M.A., Nagashima K., Sattely E.S. (2026). Identification and characterization of dietary antigens in oral tolerance. Sci. Immunol..

[33] Dong J., Ping L., Cao T., Sun L., Liu D., Wang S., Huo G., Li B. (2022). Immunomodulatory effects of the *Bifidobacterium longum* BL-10 on lipopolysaccharide-induced intestinal mucosal immune injury. Front. Immunol..

[34] Hanson K.L., Weiss A.A. (2024). Intestinal tissue response to Shiga toxin exposure. mBio.

[35] Wang Y., Guan Q.N., Zhang Z.J., Zhang Y.M. (2025). Interaction between endothelial injury and immune response in septic shock: From basic research to clinical applications. Front. Physiol..

[36] Chen Q., Chen O., Martins I.M., Hou H., Zhao X., Blumberg J.B., Li B. (2017). Collagen peptides ameliorate intestinal epithelial barrier dysfunction in immunostimulatory Caco-2 cell monolayers via enhancing tight junctions. Food Funct..

[37] Laksitorini M., Prasasty V.D., Kiptoo P.K., Siahaan T.J. (2014). Pathways and progress in improving drug delivery through the intestinal mucosa and blood-brain barriers. Ther. Deliv..

[38] Alshehade S.A., Almoustafa H.A., Alshawsh M.A., Chik Z. (2024). Flow cytometry-based quantitative analysis of cellular protein expression in apoptosis subpopulations: A protocol. Heliyon.

[39] Kari S., Subramanian K., Altomonte I.A., Murugesan A., Yli-Harja O., Kandhavelu M. (2022). Programmed cell death detection methods: A systematic review and a categorical comparison. Apoptosis.

[40] Hassan S.N., Ahmad F. (2026). Looking at the fraction with Annexin V^+^ and propidium iodide^+^: Insights into cell death types from preclinical studies in solid and haematological cancers. Apoptosis.

[41] Narayanan S., Surendranath K., Bora N., Surolia A., Karande A.A. (2005). Ribosome inactivating proteins and apoptosis. FEBS Lett..

[42] Akanyibah F.A., Zhu Y., Jin T., Ocansey D.K.W., Mao F., Qiu W. (2024). The function of necroptosis and its treatment target in IBD. Mediat. Inflamm..

